# Magnitude of mortality and its associated factors among Burn victim children admitted to South Gondar zone government hospitals, Ethiopia, from 2015 to 2019

**DOI:** 10.1186/s13052-022-01204-x

**Published:** 2022-01-21

**Authors:** Chalie Marew Tiruneh, Amare Belachew, Sileshi Mulatu, Tigabu Desie Emiru, Nigusie Selomon Tibebu, Moges Wubneh Abate, Adane Birhanu Nigat, Amsalu Belete, Belete Gelaw Walle

**Affiliations:** 1 Department of Pediatrics and Child Health Nursing, College of Health Sciences, Debre Tabor University, Debre Tabor, Ethiopia; 2 Department of Pediatrics and Child Health Nursing, School Of Health Sciences, College Of Medicine and Health Sciences, P.O. Box 79, Bahir Dar, Ethiopia; 3 Department of Adult health Nursing, College of Health Sciences, Debre Tabor University, Debre Tabor, Ethiopia; 4 Department of Psychiatry, School of Medicine, College of Health Sciences, Debre Tabor University, Debre Tabor, Ethiopia; 5 Department of Pediatrics and Child Health Nursing, College of Health Sciences and Medicine, Wolaita Sodo University, Wolaita Sodo, Ethiopia

**Keywords:** Burn injuries, Children, Ethiopia, Mortality, South Gondar

## Abstract

**Background:**

Burn is one of the leading causes of preventable death and disability every year in low and middle-income countries, which mainly affects those aged less than 15 years. Death from burn injuries carries the most significant losses, which often have grave consequences for the countries. Even though data from different settings are necessary to tackle it, pieces of evidence in this area are limited. Thus, this study was aimed to answer the question, what is the Magnitude of Mortality? And what are the factors associated with mortality among burn victim children admitted to South Gondar Zone Government Hospitals, Ethiopia, from 2015 to 2019?

**Methods:**

Institutional-based cross-sectional study design was used to study 348 hospitalized burn victim pediatrics’, from 2015 to 2019. A simple random sampling method was used. Data were exported from Epidata to SPSS version 23 for analysis. Significant of the variables were declared when a *p*-value is < 0.05.

**Result:**

The mortality rate of burn victim children in this study was 8.5% (95% CI = 5.5–11.4). Medical insurance none users burn victim children were more likely (AOR 3.700; 95% CI =1.2–11.5) to die as compared with medical insurance users, burn victim children with malnutrition were more risk (AOR 3.9; 95% CI = 1.3–12.2) of mortality as compared with well-nourished child. Moreover, electrical (AOR 7.7; 95% CI = 1.8–32.5.2) and flame burn (AOR 3.3; 95% CI = 1.2–9.0), total body surface area greater than 20% of burn were more likely (AOR 4.6; 95% CI 1.8–11.8) to die compared to less than 20% burn area and burn victim children admitted with poor clinical condition at admission were four times (AOR 4.1, 95% CI = 1.3–12.0) of mortality compared to a good clinical condition.

**Conclusion:**

The mortality among burn victim children was higher than most of the studies conducted all over the world. Medical insurance none users, being malnourished, burned by electrical and flame burn, having total body surface area burnt greater than 20%, and having poor clinical condition at addition were significantly associated with mortality of burn victim pediatrics. Therefore, timely identification and monitoring of burn injury should be necessary to prevent mortality of burn victim pediatrics.

## Background

Burn is a frequent cause of hospitalization and the most frequent injury among pediatrics patients. It is the fourth most common type of trauma worldwide, following traffic accidents, falls, and interpersonal violence. It continues to present a significant public health problem, resulting in scores of preventable deaths and disabilities every year [1].

In the Africa region, burn is a significant cause of death, and disproportionately affects those who are aged less than 15 years old [2]. Despite access to acute burn management in many healthcare facilities in Lower Middle-Income Countries (LMICs), a large majority of those suffering more severe burn worldwide are still unable to access the definitive care they need promptly [3].

The treatment of burn demands many hours for wound care from nursing staff, possibly multiple surgical procedures, and costly hospital care. This makes to have worse outcomes of injury due to lack of resources and it results in complications and death. Death from burn injuries carries the most significant losses which often have grave consequences for the countries because of higher mortality, and high years of life lost in the productive age group [4, 5].

The mortality rate of burn victim children in South Africa is 7.9% [6], in the Gambia, 21.4% [7], in Tanzania, 11.7% [8], and in Central Malawi, 27% [9]. The systematic review studies `on mortality of burn victim children reported in sub-Saharan countries was 17% [10], and East Africa, was 7.1% [11]. One of the largest burn unit hospitals in Ethiopia reported that 7.8% of burn victim children were died [12].

The extent of burn injury, causes of burn injury, depth of burn injury, age of the child, residency, condition at admission, and type of management given before and after admission were documented in different literature as a significant cause for poor outcomes of burned clients [7, 9, 13].

Burn prevention strategies should be developed by investigating the problems in LMICs including Ethiopia [14]. Since accidents are the main causes of burn injury in children, preventable strategies should aim to reduce the incidences of accidents that eventually lead to burn in developing countries, including Ethiopia [15]. There is only some study on epidemiology and outcomes of burn in the general population in Ethiopia [13, 16, 17], while very little is known about the magnitude of mortality and associated factors among burn victim pediatrics in Ethiopia. So far, no research had been conducted in our study area about burn injury. However, different observation in this area shows burn as one of the leading cause of morbidity and mortality in children. Thus, this study aimed to assess the magnitude of mortality and associated factors among burn victim children admitted to south Gondar zone public hospital.

## Methods

### Study area, design, and period

Institutional based cross-sectional study was conducted from January 1, 2015, to December 31, 2019, at public Hospitals found in south Gondar Zone, Amhara Regional State, Ethiopia. South Gondar zone is one of the zones’ in the Amhara region, which is located in the Northern part of Ethiopia. Currently, there are 8 governmental hospitals and 94 functional health centers. These hospitals and health centers serve a population of approximately 2.3 million people, from which children account for about 980,490. Among 8 hospitals, the study was conducted in four randomly selected hospitals. The average numbers of pediatric surgical bed in each hospital were fifteen. From the four selected hospital, Debre Tabor General Hospital has well organized malnutrition treatment Centers, while others have not well organized malnutrition treatment Centers.

### Study participants

All pediatrics with burn injury and admitted to south Gondar Zone public Hospitals between.

January 1, 2015, and December 31, 2019, were the target population. However, charts of burn victim pediatrics with none readable handwriting were excluded from the study.

### Sample size, and sampling technique

The sample size was determined by using the single population proportion formula with the assumptions of prevalence(P), 27% which is the proportion of mortality among pediatrics burn victims in Central Malawi [9], level of confidence (CL), 95%, the margin of error (d) =5%, $\mathrm{n}=\frac{{\left(\mathrm{Za}/2\right)}^2\mathrm{p}\left(1-\mathrm{p}\right)}{{\left(\mathrm{d}\right)}^2}=\frac{(1.96)^2\ 0.27\left(1-0.27\right)}{(0.05)^2}\kern0.75em =296.$

W Where, n = the required sample size, Zα/2 = Standard normal variation for type 1 error, p = prevalence (0.5) & d = Margin of sampling error tolerated (0.05).

The calculated sample size was 296. After considering a 15% incomplete charts, the final sample size of our study was 348.

This study was conducted in four randomly selected public health institutes in South Gondar Zone. From the beginning, a sampling frame was prepared using the patient’s medical registration number from each hospital’s registration logbook. Then, the total sample sizes were allocated proportionally for each hospital. Finally, charts of study participants were taken from each of the four selected hospitals using a computer-generated simple random sampling technique.

### Data collection tool and procedure

The data were collected and registered by using a structured checklist. The checklist was prepared by reviewing different literatures done on the same problems [8, 12, 18–21]. The checklist was focused on socio-demographic characteristics of injured children, clinical related factors, and treatment-related factors. Data were collected by four trained BSc nurses and were supervised by two physicians. Training about the objectives of the study, the contents of the tool, and data collection procedures was given for data collectors and supervisors for one day. To check the incompleteness of charts, a pre-test was conducted in 5% of the total sample size in the hospital where the area is not selected for actual data collection. The assigned supervisors and principal investigator closely monitored and supervised the whole data collection process.

### Dependent variable

#### Mortality status

##### Independent variables

Socio-demographic related factors, clinically related factors, and Management-related factors.

##### Data management and statistical analysis

The consistency and completeness of the collected data were examined during data management and analysis. Data were entered into Epi Data Version 3.1 and analysis was done using Statistical Package for Social Science (SPSS) Version 25. Frequencies and cross-tabulations were used to check for missed values of variables and to describe the study population concerning relevant variables. Furthermore, percentages, proportions, and summary statistics were used to summarize the study population characteristics. Binary logistic regression analysis was used to determine the association between the dependent and independent variables. All variables with *P*-value ≤0.25 in the bivariable analysis were included in the final model of multivariable analysis to control all possible confounders. The goodness of fit was tested by Hosmer-Lemeshow statistic and Omnibus tests. The direction and strength of association were measured by the odds ratio with 95% CI. The adjusted odds ratio along with 95% CI were estimated to identify factors associated with mortality by using multivariable analysis and finally P-value < 0.05 was considered to declare as statistically significant.

## Result

### Socio-demographic characteristics of burn victim patients

Three hundred forty-three charts were reviewed, making the response rate of 98.5%. Almost half (51.9%) of the participants were males and the age ranged from 1 year to 14 years. The majority (73.2%) of burn victims were under the age group of five years. Of the total study population, 34(9.9%) were malnourished and 36(10.5%) has different kinds of co-morbidities (Table 1).

**Table 1:** Socio demographic characteristics among Burn Victim Children Admitted to South Gondar Zone Government Hospitals, Ethiopia, from 2015 to 2019(*n* = 343)

Variables	Category	Frequency	Percentage
Sex	Male	178	51.9
	Female	165	48.1
	Total	343	100.0
Residency	Urban	106	30.9
	Rural	237	69.1
	Total	343	100.0
Age in years	< 5	251	73.2
	5–10	58	16.9
	11–14	34	9.9
	Total	343	100.0
Nutritional status	Malnutrition	34	9.9
	well nutrition	309	90.1
	Total	343	100.0
Comorbidity	No comorbidity	306	89.3
	Has comorbidity	37	10.7
	Total	343	100.0
Medical insurance user	Yes	100	29.1
	No	243	70.9
	Total	343	100

### Clinical characteristics of burn victim patients

The most frequent cause of burn was scald; it accounts for 238 (69.4%) of all burn injuries, followed by 84 of flame burn (24.5%). Electrical burn accounts for 17(4.9%) of the total injury. From 343 cases, 65(19%) burn cases had total body surface area (TBSA) burnt less than 20% and 33(9.6%) had a third-degree burn. Regarding the time of admission, Sixty-four (18.7%) of injured children presented to the hospital after 24 h of burn injury, and 114(33.2%) of burn occurred in spring (Table 2).

**Table 2:** clinical related factors among Burn Victim Children Admitted to South Gondar Zone Government Hospitals, Ethiopia, from 2015 to 2019(n = 343)

Variable	Category	Frequency	Percent(%)
Cause of burn	Scald burn	238	69.4
	Flame burn	84	24.5
	Electrical burn	17	4.96
	Chemical burn	4	1.17
Intention of injury	Accidental	335	97.7
	Homicidal	4	1.2
	Suicidal	4	1.2
	Total	343	100.0
Place of burn injury	Home	319	93.0
	Outside home	24	7.0
	Total	343	100.0
TBSA	< 20%	278	81.0
	> 20% area	65	19.0
	Total	343	100.0
Depth	first degree	42	12.2
	second degree	268	78.1
	third degree	33	9.6
	Total	343	100.0
Duration before hospital arrival	Early	279	81.3
	Late presentation	64	18.7
	Total	343	100.0
Season of injury	Spring	114	33.2
	Summer	109	31.8
	Winter	61	17.8
	Autumn	59	17.2
	Total	343	100.0
Clinical condition at admission	Poor	34	9.9
	Good	309	90.1
	Total	343	100
Infection at wound site	Yes	252	73.5
	No	91	26.5
	Total	343	100
Length of hospital stay	< 5 days	95	27.7
	5-10 days	134	39.1
	11-15 days	76	22.2
	16-20 days	34	9.9
	21-26 days	4	1.2
	Total	343	100.0

### Management related data

Of total victims, 93(27.1%) of them did not get any first aid measure before hospital arrival and only 280(81.6%) patients were received fluid resuscitation, 208 (60.%) were received antibiotic treatment and 186 (54.2%) of them were applied Nitrofurazone in the injured area. From 343 children burn victim, 29 (8.5%) (95% CI = 5.5–11.4) of them were died (Table 3).

**Table 3:** Management related factors among Burn Victim Children Admitted to South Gondar Zone Government Hospitals, Ethiopia, from 2015 to 2019 (n = 343)

Variable	Category	Frequency	Percent(%)
First aid	Yes	250	72.9
	No	93	27.1
	Total	343	100.0
Fluid at admission	Yes	280	81.6
	No	63	18.4
	Total	343	100.0
Ointment	Nitrofurazone	186	54.2
	Silver sulphadiazine	42	12.2
	TTC	39	11.4
	not applied	76	22.2
	Total	343	100.0
Antibiotics given	Yes	222	64.7
	No	121	35.3
	Total	343	100.0
Wound care frequency	TID	15	4.4
	BID	175	51.0
	Daily	153	44.6
	Total	343	100.0
Condition of patient at discharge	Improved	314	91.5
	Death	29	8.5
	Total	343	100

### Factors associated with mortality

In multivariable analysis, medical insurance, nutritional status, electrical and flame burn, TBSA burnt and clinical condition of the victim at admission was remained to show statistically significant association with mortality of burn injury.

Those burn victim pediatrics, whose families were not medical insurance users were at higher risk of mortality (AOR 3.700; 95% CI =1.2–11.5) than those who use medical insurance. Mortality was higher in malnourished burn pediatrics (AOR 3.9; 95% CI = 1.3–12.2) than well-nourished. Those burn victim pediatric population who had a poor clinical condition at the time of hospital arrival were more likely to die (AOR 4.1, 95% CI = 1.3–12.0) compared to those who present with a good clinical condition.

Pediatrics who sustained electrical burn were more likely to die (AOR 7.7; 95% CI = 1.8–32.5.2) as compared to scald injury. On the other hand, those burn victim children who sustained flame burn had a higher chance of mortality compared (AOR 3.3; 95% CI = 1.2–9.0) to scald burn. Moreover, burn victim children who had TBSA burn of greater than 20% were more likely to die (AOR 4.6; 95% CI 1.8–11.8) than those who had a burn of less than 20% TBSA (Table 4).

**Table 4:** Bivariable and multivariable logistic regression analysis among Burn Victim Children Admitted to South Gondar Zone Government Hospitals, Ethiopia, from 2015 to 2019(*n* = 3430

Variable and Category	Death		Bi-variable logistic regression analysis	Multi-variable logistic regression analysis
	Yes n (%)	No n (%)	COR With 95%CI	AOR With 95% CI
Medical insurance user				
No	24(9.9%)	219(90.1%)	2.082(0.771–5.621)	4.3(1.3–13.9)*
Yes	5(5%)	95(95%)	1	
Nutritional status				
Mal-nutrition	8(23.5%)	26(76.5%)	4.220(1.702–10.461)	3.757(1.136–12.421)*
Well-nutrition	21(6.8%)	288(93.2%)	1	
Comorbid Illness?				
Yes	5(13.9%)	31(86.1%)	1.902(0.677–5.340)	
No	24(7.8%)	283(92.2%)	1	
Clinical condition at admission				
Poor	10(29.4%)	24(70.6%)	6.360(2.660–15.204)	3.836(1.237–11.896)*
Good	19(6.1%)	290(93.9%)	1	
Duration before arrival				
Late	11(17.2%)	53(82.8%)	3.009(1.344–6.739)	
Early	18(6.5%)	261(93.5%)	1	
Cause of burn				
Chemical	1(25.0%)	3(75.0%)	5.769(0.561–59.367)	
Electrical	4(5.5%)	13(76.5%)	5.325(1.522–18.632)	7.620(1.690–33.177)*
Flame	11(13.1%)	73(86.9%)	2.608(1.120–6.073)	3.094(1.065–8.986)*
Scald	13(23.5%)	225(94.5%)	1	
The anatomical area of the burn				
Multiple	9(14.1%)	55(85.9%)	2.119(0.916–4.903)	
Single	20(7.2%)	259(92.8%)	1	
Degree Of Burn				
3rdDegree	10(30.3%)	23(69.7%)	5.652(1.409–22.677)	
2nd Degree	16(6.0%)	252(94.0%)	0.825(0.230–2.964)	
1st Degree	3(7.1%)	39(92.9%)	1	
Total body surface area				
> 20%	15(23.1%)	50(76.9%)	5.657(2.571–12.447)	4.457(1.455–13.650)*
< 20%	14(5.0%)	264(95.0%)	1	
Wound infection				
Yes	15(16.5%)	76(83.5%)	3.355(1.549–7.267)	
No	14(5.6%)	238(94.4%)	1	

*1 = Reference*

* = significant in multivariable analysis at p < 0.05

## Discussion

This study revealed that mortality related to burn injury was in line with a study conducted in East Africa (7.1%) [11], and Tanzania (11.7%) [8]. However, it is higher than studies conducted in Kenya 5% [22], Nigeria 3.8% [23], Egypt 2.5% [20], Turkey 3.8% [24], and Pakistan 2.35% [25]. The discrepancy might be explained by the lack of infrastructure, ambulance service, and health facilities in our study setting. This discrepancy might also be due to the lack of burn unit centers in our study setting, lack of trained and experienced health professionals dedicated to the care of burn patients compared to the mentioned studies [24].

Finding in this study is lower than a study conducted in Gambia (21.4%) [7], Malawi (27%) [9], and Ghana (21.3%) [26]. This discrepancy might be due to the difference in socio-demographic characteristics, and due to the reason that studies in mentioned countries were conducted in tertiary hospitals which are prone to have overflows of burn patients throughout their country and increases the rate of mortality in the hospitals [5, 9, 26]. On the other hand, in our study-setting complex and complicated burn cases are referred to another area, which might decrease the mortality rate in the study setting.

Mortality of burn victim pediatrics was more likely in medical insurance none user than being medical insurance users. This could be due to the reason that those families who did not use medical insurance may delay in seeking medical care due to costly hospital care. This is consistent with the scientific justification of the treatment of burn demands many hours for wound care from nursing staff, possibly multiple surgical procedures, and costly hospital care.

This study revealed that mortality was more likely in electrical and flame burn compared to scald burn. The latter is in line with the study done in the Gambia) [7]. On the contrary, the study conducted in Tanzania revealed the opposite result for electrical and flame burn) [8]. The discrepancy might be due to the difference in management practice, which results from the difference in the study setting between the mentioned studies. This is because all severe burn injuries including, electrical and inhalational burn-in Tanzania were admitted in multidisciplinary Intensive Care Units which have good access to management [8]. However, general and district hospitals in our setting might manage scald burn better than other causes and electrical, and flame burn were referred to other hospitals.

In this study, mortality in malnourished burn victim pediatrics was more likely, which was opposite to the study conducted in Mekelle [27]. The discrepancy might be due to the presence of a dietitian from a board of Ayder referral hospital who can recommend special feeding according to the needed calorie for the victim in addition to hospital feeding and therapeutic feeding [27]. But in most of our study settings; there is no well-organized severe acute malnutrition (SAM) unit and no dietitian in all, this is difficult to fulfill the nutritional need of the victim who had a severe catabolic response and increases basal metabolic rate after a burn injury [28, 29]. So coupled with the prior nutritional deficit, this condition can worsen the clinical condition of the victim which may end up in death [30].

Poor clinical condition at the time of admission was a significant contributor to mortality in the burn victim pediatrics population, which is in line with the study conducted in Morocco [21]. This is consistent with the scientific justification of burn victim patients who has the poor clinical condition is an indicator of extensive fluid loss, anemia, infection, increased catabolism, and/or decreased intake which all together can lead the victim to the end of his life [29].

Finding of this study revealed that mortality was more likely to burn victim children who had TBSA burn of greater than 20%, which is in line with the study conducted in Bahir Dar [31]. This is supported by the scientific evidence of those who have a larger body surface area of burn are prone to complex fluid shifts, including system-wide extravasation of fluids into unburned tissues. This results in resultant intravascular hypervolemia which can produce ischemia to body organs, lactic acidosis, and eventual cardiovascular collapse [28, 29] which increases the risk of mortality and complications.

## Conclusion

The mortality among burn victim children was higher than most of the studies conducted all over the world. Medical insurance none users, being malnourished, being burnt by electrical and flame burn, having total body surface area burnt greater than 20%, and having poor clinical condition at admission were significantly associated with mortality of burn victim pediatrics. Therefore, timely identification and monitoring of burn injury should be necessary to prevent mortality of burn victim pediatrics.

## Data Availability

The data sets used and/or analyzed during the current study are available from the Corresponding author upon reasonable request.
